# Burnout syndrome among undergraduate nursing students at a public university^1^

**DOI:** 10.1590/0104-1169.3254.2498

**Published:** 2014

**Authors:** Jamila Geri Tomaschewski-Barlem, Valéria Lerch Lunardi, Guilherme Lerch Lunardi, Edison Luiz Devos Barlem, Rosemary Silva da Silveira, Danielle Adriane Silveira Vidal

**Affiliations:** 2 Doctoral student, Universidade Federal do Rio Grande, Rio Grande, RS, Brazil. Scholarship holder from Fundação de Amparo à Pesquisa do Estado do Rio Grande do Sul (FAPERGS), Brazil; 3 PhD, Associate Professor, Universidade Federal do Rio Grande, Rio Grande, RS, Brazil; 4 PhD, Adjunct Professor, Instituto de Ciências Econômicas, Administrativas e Contábeis, Universidade Federal do Rio Grande, Rio Grande, RS, Brazil; 5 PhD, Adjunct Professor, Universidade Federal do Rio Grande, Rio Grande, RS, Brazil; 6 Master's student, Universidade Federal do Rio Grande, Rio Grande, RS, Brazil. Scholarship holder from Conselho Nacional de Desenvolvimento Científico e Tecnológico (CNPq), Brazil

**Keywords:** Students, Nursing, Burnout, Nursing Education

## Abstract

**OBJECTIVE:**

to investigate the burnout syndrome and its relationship with demographic and academic variables among undergraduate nursing students at a public university in Southern Brazil.

**METHOD:**

a quantitative study with 168 students, by applying an adaptation of the Maslach Burnout Inventory - Student Survey, validated for this study. We used descriptive and variance analysis of the data analysis.

**RESULTS:**

we found that students do not have the burnout syndrome, manifesting high average scores in Emotional Exhaustion, low in Disbelief and high in Professional Effectiveness; that younger students who perform leisure activities have greater Professional Effectiveness, unlike students in early grades with no extracurricular activities; combining work and studies negatively influenced only the Professional Effectiveness factor, while the intention of giving up influenced negatively Disbelief and Professional Effectiveness factors.

**CONCLUSION:**

the situations that lead students to Emotional Exhaustion need to be recognized, considering the specificity of their study environments.

## Introduction

The burnout syndrome has its roots in the 1970s, featuring a syndrome defined as a progressive process of emotional exhaustion and loss of professional interest, due to a prolonged period of exposure to high levels of stress arising from work situations, emerging especially among professionals who exercise care for others^(^ 1 ^)^. The occurrence of burnout among professionals comprises three multidimensional factors proposed from the Maslach Burnout Inventory: emotional exhaustion, depersonalization, and reduced personal accomplishment^(^ 1 ^)^.

In recent years, the burnout syndrome began to be investigated in college students, extending its concept and confirming the existence of the three factors derived from the Maslach Burnout Inventory, in this population^(^ 2 ^)^. Thus, the burnout syndrome among students includes: emotional exhaustion, described as the feeling of being exhausted in response to the intense demands of studying; disbelief, perceived as the development of a skeptical and distanced attitude from the study; and low professional efficacy, marked by the perception of being ineffective as students^(^ 2 ^)^.

In the environment of training for majoring in nursing, several factors may become stressors, commonly developed in full time courses, with a constantly intense pace, the pressure arising from the requirements of professors and anxiety about having a satisfactory performance in each class^(^ 3 ^)^. The characteristics of the nursing program, whose professional training is directed to care, contribute to them experiencing conflicting situations, whether in the classroom, in the laboratory, in the care for urgent / emergency cases, with the possibility of further confrontation with death, the local internship and practical classes. However, there does not seem to be a sufficient psychological preparation for coping with these situations^(^ 4 ^)^.

Entering the hospital environment and being in contact with patients with diseases, carrying out procedures causing distress, fear and trauma, power relationships with professors, inattention and negative attitudes of nursing staff in patient care, lack of integration with students from other courses in the area of health and lack of support for coping with these experiences are some examples of situations that cause stress and wear that can compromise the health and quality of life of nursing, often causing discouragement^(^ 5 ^-^ 6 ^)^.

When the students find it difficult to adapt to their own situations amid the profession, or even when not satisfied with their career choice, sources of stress and conflict can be identified, with possible repercussions for the students in their own professional future, with the environment and working relationships with the different subjects they comes to interact with, and to the care provided^(^ 7 ^)^.

All these questions seem to contribute to the difficulty in organizing the new roles and responsibilities established by the future profession, which requires care, flexibility and complexity in the care of another human being, thus starting situations of stress and emotional instability that can lead these students to the development of burnout^(^ 8 ^)^.

In Brazil, studies on burnout in nursing students^(^ 8 ^-^ 9 ^)^ showed that the students surveyed did not have the syndrome. However, these studies were conducted in private institutions, which require an investigation of burnout also among students of public institutions, in order to verify possible differences between these contexts. Still, in a qualitative study on burnout among nursing students of a public institution, different situations present in these students that may be sources of emotional exhaustion, disbelief and low professional efficacy environment were identified^(^ 10 ^)^.

At present, research on burnout in nursing students is still scarce, both nationally and internationally, only studies with students from the early undergraduate years were found^(^ 8 ^,^ 11 ^)^; final undergraduate years^(^ 9 ^,^ 12 ^)^; undergraduate students, linking stressors, burnout and the withdrawal from the course^(^ 13 ^)^; relating hardiness and burnout^(^ 14 ^)^; composing a sample of undergraduate students in the health field^(^ 15 ^)^; and a qualitative study about the manifestations of burnout among undergraduate students^(^ 10 ^)^.

Still, recognizing the experience of burnout among nurses^(^ 16 ^-^ 17 ^)^, the question is: could the experience of burnout also be present during training? Thus, considering the studies presented here, the production of current knowledge and remaining gaps in this subject, ignorance about burnout among undergraduate nursing students at a public university and its relationship with demographic and academic variables constitute the research problem.

Thus, this study is justified, since the research phenomenon still constitutes a theme rarely explored in the context in question and its recognition, as well as the identification of its possible occurrence in nursing students, are essential for coping strategies to be adopted during graduation, favoring the process of training. The objective is: to investigate the burnout syndrome and its relationship with demographic and academic variables among undergraduate nursing students at a public university in Southern Brazil.

## Method

This is a quantitative, exploratory and descriptive study, which defines characteristics and presents a profile of a particular group. Its design is characterized as cross-sectional since all measurements were done in a short period of time, providing opportunities for a description of variables and their distribution patterns.

The study was carried out with a sample of 168 undergraduate nursing students enrolled from the first to ninth grade in a public university in Southern Brazil, selected through non-probability convenience sampling. The researched undergraduate program, which began operating in 1976, had 242 students enrolled in the second half of 2011, developing into nine series since its last reformulation and third in 2005, with a total course load of 4055 hours.

The sample size was defined by a specific mathematical formula^(^ 18 ^)^, whose goal is to estimate the minimum sample size to be able to achieve certain statistical procedures, guaranteeing the reliability of the study. By knowing the total population in advance, consisting of 242 students, and applying the formula^(^ 18 ^)^, the minimum number of respondents was determined to be 150.

As a tool for data collection, an adaptation of the Maslach Burnout Inventory - Student Survey (MBI-SS) was used, original form applied to a sample of Spanish students^(^ 2 ^)^ and translated into Portuguese in a survey of college students^(^ 5 ^)^. The instrument has three subscales: Emotional Exhaustion, Disbelief and Professional Effectiveness, and was answered on a seven-point Likert frequency scale, using 0 for "never" to 1 "once a year or less," 2 to "once a month or less", 3 for "a few times a month," 4 for "once a week", 5 for "a few times a week" and 6 for "every day". Thus, the burnout syndrome manifests when the student obtains high scores on Emotional Exhaustion and Disbelief, associated with low scores on Professional Effectiveness^(^ 5 ^)^.

For this research, an instrument with 21 questions was elaborated, containing fifteen questions of the instrument translated into Portuguese^(^ 5 ^)^, of which five had their writing adapted and six new questions were prepared, considering the participation in practical classes and traineeships of undergraduate students in nursing. The 21 questions were subject to construct validation through factor analysis, seven were excluded from the instrument because of presenting low factor loadings (less than .40), high factor loadings (greater than 0.40) by more than a factor, not presenting conceptual coherence with the proposed block or even form isolated blocks (formed by a single question). Among the excluded questions, two (2) were from the instrument translated into Portuguese, one (1) referred to the adaptation of the instrument's writing and four (4) proposals were new issues. The application of factor analysis permitted grouping the 14 questions on three constructs for the three factors of burnout experienced in everyday undergraduate nursing students: Emotional Exhaustion, Disbelief and Professional Effectiveness. Still in relation to the construct validation, the instrument's Cronbach's alpha showed a value of 0.72, while the coefficients of the three factors were between 0.72 and 0.78, which proves the reliability of the three-factor instrument.

The instrument also presented an initial part to characterize the subjects, containing socio-demographic and academic characteristics that allowed the association of burnout variables such as age, sex, marital status, children, current occupation^(^ 8 ^,^ 15 ^,^ 19 ^)^, whom the respondent resides with^(^ 8 ^)^, year of enrollment in the course, grade level, satisfaction with the course, work experience in healthcare, having another higher education, leisure activities^(^ 15 ^,^ 19 ^)^, and intention to leave the course^(^ 7 ^,^ 13 ^,^ 15 ^,^ 19 ^-^ 20 ^)^.

Data collection was conducted in the classroom, between October and November 2011, by two previously trained undergraduate research fellows. The instrument was delivered, after authorization from the professors, being answered by students and collected soon after, along with the signed Statement of Consent.

The results concerning the study sample were obtained by means of descriptive statistics, by using the means and frequency distribution of the constructs and their indicators; and analysis of variance (ANOVA) between the different groups of respondents, according to characteristics of the sample, to check for possible significant differences. For data analysis, the statistical software SPSS (Statistical Package for Social Sciences) version 17.0 was used, facilitating the process of organizing data into tables for the sake of better visualization of the results and their interpretation.

This study, complying the recommendations of National Health Council Resolution No. 196/96, was submitted to the local Research Ethics Committee and received a favorable opinion (Opinion 135/2011).

## Results

With regard to the socio-demographic data of the sample, the 168 subjects have an average of 24.5 years, with 23 years for the students of the lower grades (1st to 4th grade) and 25 of the final grades (5th to 9th grade). Most subjects are female (92.9%) are single (86.3%), have no children (86.9%) do not work (84.5%) and live with their parents (51.2%). With regard to the academic characteristics, 79.3% of subjects have chosen nursing as the first option, with superficial knowledge of the course before entry (47.6%). Still, most subjects performed extracurricular activities (73.2%), consider that they have a suitable location for their studies (83.9%) and have a good relationship with colleagues (82.7%). They are pleased with the ongoing course (91.1%) and do not express or have expressed intention of ever giving up (67.3%).

Concerning the evaluation results of the burnout factors (Table 1), the descriptive analysis has shown that the Emotional Exhaustion factor had the highest average of the instrument (4.00), indicating the existence of depletion in students, especially at the end of the day when they had class, whether theoretical or practical or laboratory or internship activities, as well as when they rise to face another day of school, in the classroom or lab. These situations are faced with an average frequency close to "once a week". In the Disbelief factor, the corresponding mean was 1.80, indicating that students feel distant and skeptical of their studies with a "once a month or less" frequency. The Professional Effectiveness factor showed a value of 4.54, indicating that students realize they are being effective in their studies, especially for learning many interesting things during the course, and consider themselves good students, with a frequency close to "a few times a week".

**Table 1:** Average frequency of burnout experienced by students. Rio Grande, Brazil, 2012

Factors		N	Frequency
Emotional Exhaustion		168	(4.00)
	q-01 I feel emotionally drained by my studies	168	3.71
	q-04 I feel exhausted at the end of a day that I have class (classroom and laboratory)	168	4.56
	q-05 I feel exhausted at the end of a day that I practice / internship activities in health institutions	168	4.11
	q-08 I feel tired when I wake up to face another day of school (classroom and laboratory)	168	4.00
	q-16 I am consumed by my studies	168	3.64
Disbelief		168	(1.80)
	q-02 I question the meaning and the importance of my studies	168	2.61
	q-13 I have become less interested in studies since I joined this university	168	1.27
	q-14 I have become less interested in my studies	168	1.43
	q-19 I have been more skeptical of my potential and the usefulness of my studies	168	1.90
Professional effectiveness		168	(4.54)
	q-03 I have learned many interesting things in the course of my studies	168	5.32
	q-06 During class (lecture and lab room), I feel confident: I perform tasks effectively	168	4.44
	q-15 I consider myself a good student	168	4.51
	q-20 I believe that I am effective in contributing to classes (classroom and lab room) I attend	168	4.05
	q-21 I believe that I am effective in contributing to practical activities / internships in health institutions I attend	168	4.36

The ANOVA (Table 2) allowed us to analyze the possible differences in the averages of the burnout factors in undergraduate nursing students, considering their demographic and academic characteristics. With regard to the relationship between the factors of burnout and socio-demographic variables, significant differences were observed for the age and use of leisure activities variables, both in the Professional Effectiveness factor. Younger students who perform leisure activities have a higher perception of their Professional Effectiveness. Significant differences were also identified between the work variable and the three factors of burnout, noting that students that combine work and study, have lower averages in Emotional Exhaustion, Disbelief and Professional Effectiveness. Unlike what happened in the Emotional Exhaustion and Disbelief factors, balancing work and studies negatively influenced the Professional Effectiveness factor, demonstrating that students who work and study consider themselves less effective in their studies compared with the students who only study.

**Table 2:** Relationship between burnout and sociodemographic factors and academic variables. Rio Grande, Brazil, 2012

Factors		Emotional exhaustion			Disbelief			Professional Effectiveness	
		m	p		m	p		m	p
Age									
	≤ 25 years (n=115)	-	-		-	-		4.64	0.042*
	> 25 years (n=53)							4.28	
Works									
	Yes (n=26)	3.34	0.010*		1.22	0.014*		4.12	0.026*
	No (n=142)	4.11			1.88			4.59	
Performs leisure activity									
	Yes (n=135)	-	-		-	-		4.63	0.009*
	No (n=33)							4.12	
Current Grade									
	1st – 4th grade (n=78)	-	-		-	-		4.37	0.050*
	5th – 9th grade (n=90)							4.67	
Has extracurricular activity									
	Yes (n=123)	-	-		-	-		4.63	0.041*
	No (n=45)							4.27	
Intention to withdraw from the course									
	Yes (n=55)	-	-		2.22	0.005*		4.26	0.016*
	No (n=113)				1.6			4.67	

* Significant difference at 5%

In the relationship between burnout and academic variables, it was found that students from early grades and students who do not perform extracurricular activities had lower means in Professional Effectiveness students of final grades and extracurricular activities they perform. The intention to withdraw from the course variable showed significant relationship with Disbelief and Professional Effectiveness factors. It was found that subjects who have expressed the intention to withdraw from the course have a higher sense of disbelief regarding studies and lower perception of their Professional Effectiveness.

## Discussion

With respect to the averages of the burnout factors in the studied sample, considering the frequency range 0-6, Emotional Exhaustion was rated high; Disbelief and Professional Effectiveness were low and high, respectively. Thus, no indicators for burnout were noted in the sample studied, since the criteria for the presence of burnout suggest high averages in emotional exhaustion and low in Disbelief and Professional Effectiveness^(^ 2 ^,^ 5 ^)^.

It is noteworthy that the results for the Emotional Exhaustion factor differ from other research with students in the area of health^(^ 15 ^)^ and graduate students in nursing from the initial grades^(^ 8 ^)^ and the final ones^(^ 9 ^)^, where average rates were identified for this factor, therefore, they were less expressive than those found in the present study. The theoretical model for the development of burnout^(^ 21 ^)^ suggests, however, that emotional exhaustion is the first dimension to manifest, followed by elevation of Disbelief and therefore the sense of low Professional Effectiveness, which suggests that burnout may be developing in the students investigated, as found in research with students in the area of health^(^ 15 ^)^.

As the interrelationship between psychological variables, personality, stress, coping and burnout in nursing students is investigated, it has been considered that the completion of the undergraduate degree in nursing can lead to increased levels of stress and burnout^(^ 12 ^)^ given the specificities of the situations nursing students experience.

The relationship between the age of the students and the Professional Effectiveness factor indicates that the younger ones have a higher sense of efficacy in relation to their studies, which contradict the results of other research with nursing students in the final years of graduation^(^ 9 ^,^ 12 ^)^, students in the area of health^(^ 19 ^)^ and nursing residents^(^ 22 ^)^, which showed that individuals with higher age seem to have more confidence in carrying out their activities and view more realistically their efforts and achievements. Whereas the centrality of nursing lies in the care of others, constituting a justification of this career option for many^(^ 7 ^)^, it is possible that a more idealized view is identified in younger students.

Maintaining simultaneous work activities along with the course was associated with three factors of burnout, with lower averages in the three factors negatively influencing only the Professional Effectiveness factor. Thus, students who do not yet carry out any professional activity may have greater Disbelief regarding studies, since they possibly experience greater doubts and questions regarding the application of theoretical knowledge in practical activities and internships, which may lead them to distance themselves from the studies^(^ 4 ^)^. However, students who reconciled work and studies had lower perception of Emotional Exhaustion, not confirming that maintaining work simultaneous with course activities may compromise the health and quality of life of students, because of the excessive fatigue and wear such situations create^(^ 6 ^)^. It is possible that the experiences and learning from work carried out contribute to the strengthening of emotional coping of students in training activities, and consequently to a lower perception of Emotional Exhaustion.

It is noteworthy that the accomplishment of leisure activities was positive towards greater professional effectiveness, since it can help communication, interpersonal relationships, as well as the relief of tension and students feel more confident and effective in carrying out their activities^(^ 23 ^)^.

Regarding the grades, the beginning of the academic course had lower feelings of Professional Effectiveness. In the initial grades, most of the workload is developed with content from the Biological and Health Sciences and the Humanities and Social Sciences, while the disciplines of Nursing Sciences have a smaller workload. This distribution of the contents of the different sciences seems to not sufficiently contribute to the knowledge of what the work of nurses in different health institutions is^(^ 7 ^)^, which may contribute to the student not realizing the importance and practical application of their studies, possibly compromising their sense of professional efficacy.

A similar situation is presented to students who do not develop extracurricular activities, since these also had lower Professional Effectiveness. Conducting extracurricular activities during training allows the student to view and enter a more comprehensive and participatory of reality, full of conflicts and contradictions, contributing to strengthen their confidence in performing activities related to their professional option^(^ 24 ^)^, recognizing the importance of their actions.

In the relationship evidenced between Disbelief and Professional Effectiveness factors and the variable intention to withdraw from the course, similarly to our findings, research with students in the area of health found that those who expressed interest in quitting the course showed a higher sense of disbelief regarding studies and less Professional Effectiveness^(^ 15 ^)^., by expressing dissatisfaction with their course, the students possibly do not realize the meaning and the reward of their effort in carrying out training activities, which implies greater wear when performing their tasks, emerging attitudes of skepticism and distance in relation to their studies and little development effectiveness of its actions. Thus, the withdrawal of the course has been understood as a consequence of the burnout process^(^ 15 ^,^ 19 ^)^.

The little knowledge of students about their chosen course, or even the lack of interest in the chosen profession, can contribute to feelings of dissatisfaction, frustration, triggering stressful situations, which seems to motivate undergraduate students in nursing their wish to give up or even to give up the course^(^ 7 ^)^. The withdrawal from the nursing course, troubling to educational institutions seems to be related to the recognition of low status and the devaluation of the profession, the lack of professional autonomy when compared to other professions in the health field; school failure, failures, unmet expectations of students by the institutions; personal reasons, such as changes or financial problems, and especially difficult to deal with stress, including from the practical classes and internships^(^ 7 ^,^ 13 ^)^.

In a study of newly graduated nurses, it was found that the presence of high levels of burnout was accompanied by the desire to give up the profession and the development of burnout can be previously identified by expressions of lack of interest in studies in the last year of graduation^(^ 20 ^)^. In this sense, the development of burnout among nursing students can be associated to specific situations of the university environment; however, can also be linked to the activities of professional practice exercised by students, signaling the importance of interventions that focus on elements that may be associated with burnout in students^(^ 19 ^,^ 25 ^)^, thus favoring the professional education process.

## Final Considerations

This study showed that the undergraduate students in nursing from the sample did not show the burnout syndrome, however, showed high scores on Emotional Exhaustion factor, which may constitute an early indicator of the development of the burnout process. High levels of emotional exhaustion experienced by academics reinforce the need for ongoing investigations, reflections and discussions in educational institutions, focusing on situations and experiences that may contribute to wear and exhaustion in undergraduate students in nursing, considering especially the specificity of their training environments.

Noteworthy is the association identified between the intention to quit and Disbelief and Professional Effectiveness factors, requiring follow-up actions aimed at strengthening the identity with the profession and the needs of academics who are in a position of questioning their career choice.

The limitations of this research are: we emphasize that, since it was conducted in a specific population of students at a public university in Southern Brazil, the results cannot be generalized. The study indicates the need for further studies on burnout among undergraduate students in nursing, paying attention to the recognition of their training environments, in view of the relevance of identifying, among others, particularities of the context of nursing education that may be associated with the increase in the mean Emotional Exhaustion scores.
